# HTLV infection in persons with sexually transmitted diseases in Spain

**DOI:** 10.3389/fimmu.2023.1277793

**Published:** 2023-12-07

**Authors:** Oskar Ayerdi, Rafael Benito, Diego Ortega, Antonio Aguilera, Natalia Montiel, Ilduara Pintos, Alberto Díaz de Santiago, Begoña Baza, Vicente Soriano, Carmen de Mendoza

**Affiliations:** ^1^ Sexually transmitted Infections Clinic, Centro Sanitario Sandoval, Madrid, Spain; ^2^ Microbiology Department, Hospital Clínico Universitario Lozano Blesa, Zaragoza, Spain; ^3^ Microbiology Department, Hospital Miguel Servet, Zaragoza, Spain; ^4^ Microbiology Department, University of Santiago, Santiago de Compostela, Spain; ^5^ Microbiology Department, Hospital Universitario Puerta del Mar, Cádiz, Spain; ^6^ Internal Medicine Laboratory, Puerta de Hierro University Hospital and Research Foundation-IDIPHISA, Madrid, Spain; ^7^ Public Health Unit, UNIR Health Sciences School and Medical Center, Madrid, Spain

**Keywords:** HTLV-1, sexually transmitted infections, syphilis, HIV, transmission

## Abstract

**Background:**

HTLV-1 infection is a neglected disease, despite estimates of 10 million people infected worldwide and producing life-threatening illnesses in 10% of carriers. Sexual transmission is the main route of contagion. However, HTLV-1 is not listed among sexually transmitted infections (STIs).

**Methods:**

Serum from all consecutive individuals who had attended six STI clinics across Spain during the last 12 months were tested for HTLV antibodies using a commercial enzyme immunoassay (EIA). Reactive samples were confirmed by immunoblot.

**Results:**

A total of 2,524 samples were examined. The majority (1,936; 76.7%) belonged to men, of whom 676 (34.9%) were men who have sex with men (MSM) receiving HIV pre-exposure prophylaxis. Although native Spaniards predominated (1,470; 58.2%), up to 593 (23.5%) came from Latin America and 139 (5.5%) were African. A total of 26 individuals were initially EIA reactive and immunoblot confirmed 5 as HTLV-1 and 7 as HTLV-2. All but one HTLV-1+ case came from Latin America. Three were men and two were women. Among Latin Americans, the HTLV-1 seroprevalence was 0.67%. In contrast, all seven HTLV-2+ were native Spaniards and former injection drug users, and all but one were HIV+.

**Conclusion:**

The rate of HTLV infection among individuals with STIs in Spain is 0.5%, which is greater than in the general population. These results support the introduction of universal HTLV screening in persons who attend clinics for STIs.

## Introduction

HTLV-1 infection is a neglected disease, despite infecting around 10 million people worldwide (1, 2) and producing life-threatening illnesses in roughly 10% of carriers (3). HTLV-1 infection is highly endemic in parts of Latin America, Equatorial Africa, and Southern Japan (4, 5). Sexual transmission is the most frequent mechanism of contagion (6). Transmission may also occur following parenteral exposure among injection drug users or recipients of contaminated blood or solid organ transplants (7, 8). In endemic regions, vertical transmission of HTLV-1 from mother to child, especially throughout breastfeeding, is also common (9).

Globally, more than 80% of new HTLV-1 infections are estimated to be acquired during adulthood (10, 11). Sexual transmission is more frequent from men to women than vice versa (12). Neutralizing antibodies and a weak local immune response to the infection most likely explain the limited ability of HTLV transfer via cervicovaginal secretions (13). In contrast, the seminal fluid milieu enhances HTLV replication (14). Despite all these facts, HTLV-1 is not listed among sexually transmitted infections (STI) (15). Information on the relative risk of HTLV-1 transmission in persons with multiple sexual partners or in men who have sex with men (MSM) is scarce (16, 17). Herein, we report the results of the first large cross-sectional survey of HTLV antibodies in consecutive individuals who attended clinics for STIs in Spain.

## Methods

A nationwide HTLV-1 register was created in Spain in 1989. The main demographics, clinical symptoms/signs, and laboratory findings are collected for each new HTLV-1 case at baseline and longitudinally using a standardized case report form. A biological repository of specimens stores patient samples when possible. Members of the Spanish HTLV Network cover most of the lab facilities nationwide where this virus can be diagnosed, including public or private microbiology labs or blood banks (18). From the coordination center, all members of the network were invited to participate in the current study during the year 2021.

Serum specimens collected from consecutive individuals who attended six clinics across Spain and with a diagnosis of at least one STI during the last 12 months were tested for HTLV antibodies. A commercial enzyme immunoassay (EIA) was used for screening for HTLV-1/2 antibodies (HTLV Abbott). The sensitivity and specificity of this test have been evaluated previously and are considered reliable for screening purposes (19). Reactive samples were then confirmed by immunoblot (Inno-Lia HTLVI/II, Fujirebio). Given its good performance and easy interpretation, this test has replaced classical Western blot assays as confirmatory and discriminatory of serum HTLV-1 and HTLV-2 antibodies (20). Demographics (age, gender, and country of origin) and STI diagnoses were recorded for all patients.

### Statistical analysis

Figures are given in absolute numbers and percentages. Quantitative and qualitative variables are described as medians with interquartile ranges, means with standard deviations, or proportions. Bivariate comparisons of quantitative variables were performed using the Chi^2^ test.

All statistical analyses were performed using the IBM SPSS package for Windows v25.0 (IBM Corp, Armonk, NY). All tests were two-tailed, and only p-values <0.05 were considered significant.

### Ethical approval

The study was designed as a multicentre and retrospective collection of anonymized and consecutive clinical data associated with serum HTLV-1 antibodies. It was approved by the Puerta de Hierro University Hospital ethics committee (ref. PI 185/21).

## Results

A total of 2,524 samples were analyzed. The majority (1,936; 76.7%) belonged to men, of whom, 676 (34.9%) were MSM receiving HIV pre-exposure prophylaxis (PrEP). The median age of the study population was 37 (IQR, 18) years old. Although native Spaniards predominated (1,470; 58.2%), up to 593 (23.5%) came from Latin America and 139 (5.5%) were Africans. The most prevalent STIs diagnosed within the last 12 months were syphilis (43.2%), *gonorrhea* (20%), HIV (19.7%), *Chlamydia trachomatis* (17.9%), and hepatitis B (8.6%).

A total of 26 individuals were initially reactive for HTLV antibodies on the EIA screening. Using immunoblot, 5 were confirmed as HTLV-1 and 7 as HTLV-2, 2 were indeterminate, and 12 were negative.

The overall seroprevalence for HTLV-1 was 0.2% and for HTLV-2 was 0.28%. As shown in **Table 1**, all but one of the HTLV-1-positive patients came from Latin America (2 Colombia, 1 Brazil, and 1 Peru). Three were men and two were women. All men were MSM, of whom two were HIV-positive and another was under PrEP. The only native Spaniard was a 77-year-old woman who had been tested for HTLV-1 following a diagnosis of hepatitis B. The most frequent STIs, along with HTLV-1, in these five cases were syphilis (3), HIV-1 (2), *Chlamydia trachomatis* (2), and hepatitis B (1). Among Latin Americans with STIs, the HTLV-1 seroprevalence was 0.67%.

**Table 1:** Main features of individuals with HTLV infection and sexually transmitted diseases in Spain.

No.	HTLV type	Sex	Age (years-old)	Risk behavior	Country of birth	STI
1	HTLV-1	M	43	MSM	Colombia	Syphilis, HIV, *C. trachomatis*
2	HTLV-1	F	65	Multiple sex partners	Colombia	Syphilis
3	HTLV-1	M	26	MSM, sexual worker	Brazil	Syphilis, HIV
4	HTLV-1	M	33	MSM, PrEP	Peru	Gonorrhea
5	HTLV-1	F	77	?	Spain	HBV
6	HTLV-2	M	57	IDU	Spain	Syphilis
7	HTLV-2	M	50	IDU	Spain	HIV
8	HTLV-2	M	52	IDU	Spain	HIV, HCV
9	HTLV-2	M	51	IDU	Spain	HIV, HCV
10	HTLV-2	M	54	IDU	Spain	HIV, HCV
11	HTLV-2	M	51	IDU	Spain	HIV, HCV, Gonorrhea
12	HTLV-2	F	52	IDU	Spain	HIV

M, male; F, female; MSM, men who have sex with men; IDU, injection drug user; HBV, hepatitis B virus; HCV, hepatitis C virus.

All seven HTLV-2 cases were native Spaniards and all had been injection drug users. The most frequent STIs diagnosed within the last 12 months, along with HTLV-2, were HIV-1 (6), hepatitis C (4), *Neisseria gonorrhoeae* (1), and syphilis (1). The overall HTLV-2 seroprevalence among native Spaniards with STIs was 0.48%.

## Discussion

A rebound in STIs following the relief of social isolation measures taken to curb the COVID-19 pandemic has been noticed worldwide (21, 22). Even before, STIs had been on the rise along with a loss of fear of acquiring HIV, as a compensatory effect of the widespread use of PrEP (23–26). More than 400 million new STIs occur each year worldwide. Four agents are the predominant etiologies, namely syphilis, gonorrhea, chlamydia, and trichomoniasis. However, there are many more, including HIV, papillomavirus, herpes simplex, hepatitis B, etc.

Despite infecting roughly 10 million people worldwide and causing neurological disease and/or lymphoproliferative disorders in 10% of carriers, HTLV-1 infection remains a neglected disease (2). In endemic regions of Japan, following the implementation of HTLV-1 antenatal screening, vertical transmission decreased drastically. However, HTLV-1 infection in adolescents and adults through sexual contact has replaced vertical transmission as the main route for transmission. In Japan, which has a population of 125 million, roughly 3,000 new HTLV-1 infections occur annually (27).

Persons engaged in high-risk HIV behaviors who receive antiretrovirals to prevent HIV acquisition are recommended to be tested periodically for distinct STIs. Specifically, certain MSM groups have fueled outbreaks of hepatitis A (28), hepatitis C (29), and more recently, mpox (30). When acute infections are symptomatic, the etiology can be excluded and preventative measures implemented. However, when acute infections are asymptomatic, following HTLV-1 exposure, new carriers may establish a silent population reservoir from which the virus can spread unnoticed.

Although HTLV-1 is nowadays a predominantly sexually transmitted agent, testing is not mentioned in HIV pre-exposure guidelines (31). A recent study conducted in England examined the rate of HTLV infection in this population. Although none of the 2015 HIV PrEP users were infected, the authors cautioned that up to 75% were men of white ethnicity born in Europe. Hence, persons from HTLV-1 endemic regions were poorly represented (32).

In our survey, we examined 2,524 individuals attending STI clinics in Spain, and we found five cases of HTLV-1 and seven cases of HTLV-2 infection. All HTLV-1 male cases were young Latin Americans. The two women were 65 years old or older. One was a former Latin American sex worker. Another was an older Spaniard woman for whom we could not find any potential source of HTLV-1 infection. Two of the HTLV-1-positive men were coinfected with HIV-1. Both acknowledged multiple sexual partners, some of whom were using PrEP. The possibility of the use of antiretrovirals to protect against both HIV and HTLV infections warrants further consideration (33). At this time, it is unclear to what extent the use of antiretrovirals to treat or prevent HIV contagion might protect against sexual transmission of HTLV-1 infection (34). *In vitro* data support that cabotegravir may halt HTLV-1 transmission (35) and long-acting formulations of this drug are increasingly being used by persons engaged in high-risk sexual behaviors. Our findings highlight the convenience of adding anti-HTLV testing to the screening of individuals presenting with or at risk of any STI.

All seven HTLV-2-positive individuals identified in our STI study were native Spaniards and former injection drug users. Moreover, all but one were HIV-positive and four had hepatitis C. Several studies in Europe and North America have highlighted the occurrence of a large-scale spread of HTLV-2 during the 1980s and 1990s among injection drug users (36, 37). This explains the frequent identification of coinfection with HIV and hepatitis C. Interest in HTLV-2, however, has been lower than in HTLV-1 because evidence to date has only occasionally demonstrated human pathogenicity, with only anecdotal cases of myelopathy or neuromuscular disease (38, 39).

Our study has several limitations. First, we should acknowledge that 14 out of 26 initially reactive EIA specimens were disregarded since HTLV immunoblot was either negative (n=12) or indeterminate (n=2). We did not test these samples using molecular techniques such as polymerase chain reaction. Prior studies have unveiled HTLV-2 proviral gene sequences mainly in HIV-coinfected individuals (40, 41). Therefore, our figures for HTLV-2 could underestimate to some extent the real number of HTLV-2 infections in our study population. Second, individuals tested in our study population should not be considered representative of the STI population in Spain, as we included a disproportionate number of MSM and of the subset under PrEP. However, in our experience, most of the individuals who attend STI clinics in Spain are engaged in high-risk practices for HIV and are familiar with the healthcare system. In contrast, other individuals occasionally presenting with gonorrhea and/or syphilis are generally attended to by general practitioners. Accordingly, this group escaped our testing, which focused on those who attend STI clinics.

Estimates from hospital discharges (42), clinical presentation (43), and the HTLV national register (18) have concluded that HTLV-1 is an orphan disease in Spain, meaning that it affects less than 5 per 10,000 citizens. In our study, the overall seroprevalence of HTLV infection among individuals with STIs in Spain was 0.5%, significantly greater than in the general population. The rate of HTLV-1 infection among individuals with STIs from Latin America approached 0.7%. Altogether, our results support the introduction of universal HTLV screening in all individuals who attend clinics for STIs in Spain. It would be worth collecting information from similar studies conducted in other non-endemic countries with significant migrant flows from HTLV-endemic regions.

## Data availability statement

The original contributions presented in the study are included in the article/supplementary material, further inquiries can be directed to the corresponding author/s.

## Ethics statement

The studies involving humans were approved by the Ethics committee of Puerta de Hierro University Hospital ref. PI 185/21. The studies were conducted in accordance with the local legislation and institutional requirements. The participants provided their written informed consent to participate in this study. Written informed consent was obtained from the individual(s) for the publication of any potentially identifiable images or data included in this article.

## Author contributions

VS: Conceptualization, Formal analysis, Funding acquisition, Methodology, Supervision, Writing – original draft. OA: Investigation, Writing – review & editing. RB: Data curation, Writing – review & editing. DO: Data curation, Writing – review & editing. AA: Data curation, Writing – review & editing. NM: Formal analysis, Writing – review & editing. AD: Formal analysis, Writing – review & editing. BB: Data curation, Writing – review & editing. CM: Data curation, Funding acquisition, Investigation, Methodology, Writing – review & editing. IP: Data curation, Writing – review & editing.

## Group Members of the Spanish HTLV Network

E. Calderón (Hospital Virgen del Rocío & CIBERESP, Sevilla); M. Rodríguez-Iglesias, N. Montiel & T. Trujillo (Hospital Universitario Puerta del Mar, Cadiz); I. Viciana (Hospital Virgen de la Victoria, Málaga); T. Cabezas (Hospital Torrecárdenas, Almería); A. Lozano, E. Fernández-Fuertes & J.M. Fernández (Hospital de Poniente, Almería); F. Garcia and M. Alvarez (Hospital Universitario Clínico San Cecilio, Granada); R. Benito, S. Algarate & M. Ducons (Hospital Clínico Universitario Lozano Blesa, Zaragoza); D. Ortega (Hospital Miguel Servet, Zaragoza); C. Cifuentes, V. Fernández-Baca & J.V. Fernández-Montero (Complexo Hospitalario Universitario de Santiago-CHUS, Santiago de Compostela); M.D. Maciá (Hospital Son Espases, Mallorca); A. Hernández-Betancor & A.M. Martín (Hospital Insular Hospital Universitario, Las Palmas de Gran Canaria); M.J. Pena (Hospital Universitario Dr. Negrín, Las Palmas de Gran Canaria); M. Hernández, A.M. López-Lirola & J.L. Gómez-Sirvent (Hospital Universitario La Laguna, Tenerife); R. Copado (Hospital Dr. José Molina Orosa, Lanzarote); M.E. Cano (Hospital Universitario Marqués de Valdecilla, Santander); S. Rojo (Hospital Clínico Universitario, Valladolid); J.M. Eirós (Hospital Rio Hortega, Valladolid); M. Rodríguez (Hospital del Bierzo, Ponferrada); C. Gómez-Hernando (Complejo Hospitalario Virgen de la Salud, Toledo); A. González-Praetorius (Hospital Universitario, Guadalajara); A. Rando (Hospital Vall d’Hebrón, Barcelona); L. Force (Hospital General, Mataró); E. Miró (Hospital Santa Creu i Sant Pau, Barcelona); A. Cebollero (Clilab Diagnostics, Barcelona); J.F. Delgado (Hospital Universitario Parc Taulí, Sabadell); G. Rodríguez & L. Fernández-Pereira (Hospital San Pedro de Alcántara, Cáceres); A. Aguilera & S. Pereira (Hospital Conxo-CHUS, Santiago); J. García and R. Arcay (Complexo Hospitalario de Ourense); M. Trigo, J. Diz & M. García-Campello (Complejo Hospitalario, Pontevedra); S. Cortizo, S. Pérez & L. Morano (Hospital do Meixoeiro, Vigo); G. Reina (Clínica Universidad de Navarra, Pamplona); M. Arazamendi & Y. Salicio (Hospital Donostia, San Sebastián); E. Ugalde, M.C. Nieto & P. Liendo (Hospital Universitario de Basurto); A.J. Goikoetxea (Hospital Universitario de Cruces, Baracaldo); M.D. Ocete (Hospital General Universitario, Valencia); J.M. Ramos & I. Escribano (Hospital Universitario, Alicante); S. Sauleda & M. Pirón (Banco de Sangre & Tejidos, Barcelona); R. González, A. Richart & L. Barea (Centro de Transfusiones, Madrid); A. Jiménez & L. Blanco (Centro de Hemoterapia y Hemodonación de Castilla y León, Valladolid); L. Navarro (Centro de Transfusiones de la Comunidad Valenciana, Valencia); O. Ayerdi, B. Baza, C. Rodriguez & J. del Romero (Centro Sanitario Sandoval, IdISSC, Madrid); A. Galar, T. Aldamiz, M. Valeiro & L. Pérez (Hospital Gregorio Marañón, Madrid); I. Rodríguez-Avial (Hospital Clínico San Carlos, Madrid); L. Martín-Carbonero (Hospital Universitario La Paz, Madrid); M. Fernández-Ruiz, P. Parra, N. Redondo & T. Ruiz-Merlo (Hospital Universitario 12 de Octubre & CIBERINFEC, Madrid); M.J. Pozuelo (Universidad CEU-San Pablo, Madrid); P. Barreiro (Enfermera Isabel Zendal Emergency Hospital, Madrid); A. Treviño, F. de Jesús, O. Corral & V. Soriano (UNIR Health Sciences School, Madrid); I. Pintos, V. Moreno-Torres, M. Blanco, A. Huertas, J.A. Vargas-Núñez & C. de Mendoza (IIS Hospital Universitario Puerta de Hierro, Majadahonda).
